# Clinical course of sinus node dysfunction after thoracoscopic surgery for atrial fibrillation—analysis of the Atrial Fibrillation Ablation and Autonomic Modulation via Thoracoscopic Surgery (AFACT) study

**DOI:** 10.1007/s10840-020-00722-0

**Published:** 2020-03-14

**Authors:** Jolien Neefs, Shaëlle A. Ons, Wouter R. Berger, Sébastien P. J. Krul, Nicoline W. E van den Berg, Femke R. Piersma, Marcel A. M. Beijk, WimJan P. van Boven, Antoine H. G. Driessen, Joris R. de Groot

**Affiliations:** 1 Department of Cardiology, Heart Center, Amsterdam UMC, University of Amsterdam, Amsterdam, The Netherlands; 2 Department of Cardiothoracic Surgery, Heart Center, Amsterdam UMC, University of Amsterdam, Amsterdam, The Netherlands

**Keywords:** Surgical ablation, Atrial fibrosis, Ganglion plexus, Thoracoscopic surgery, Pulmonary vein isolation

## Abstract

**Purpose:**

Sinus node dysfunction (SND) may complicate thoracoscopic surgical atrial fibrillation (AF) ablation. Identifying patients at risk is important, as SND may require temporary or permanent pacing. To determine the incidence of postoperative SND and duration of symptoms in patients who underwent thoracoscopic surgical ablation.

**Methods:**

Patients with paroxysmal or persistent AF included in the Atrial Fibrillation Ablation and Autonomic Modulation via Thoracoscopic Surgery (AFACT) study underwent pulmonary vein isolation and additional left atrial ablations on indication. Patients were randomized to ganglion plexus ablation or control. SND was defined as symptomatic or asymptomatic junctional rhythm exceeding sinus rate within 30 days postoperatively. The SND risk was assessed by using a univariable logistic regression model. The rate of pacemaker implantation was determined.

**Results:**

The AFACT study included 240 patients. SND developed in 17 (7.1%) patients, not affected by randomized treatment, *p* = 0.18. SND patients more often had persistent AF (88.2%) than patients without SND (57.4%), *p* = 0.01. After univariable testing, persistent AF (OR 5.57 CI 1.52–35.90, *p* = 0.02) and additional left atrial ablations (OR 12.10 CI 2.40–220.20, *p* = 0.02) were associated with postoperative SND. Six (35.3%) patients needed temporary pacing for 1–7 days; permanent pacemakers (PMs) were implanted for SND in five (29.4%) patients.

**Conclusion:**

Additional left atrial ablations strongly increase the SND risk. The majority of SND was temporary, and sinus rhythm resolved within days, which indicates that a conservative approach with regard to pacemaker implantation should be considered.

**Electronic supplementary material:**

The online version of this article (10.1007/s10840-020-00722-0) contains supplementary material, which is available to authorized users.

## Introduction

In patients with symptomatic atrial fibrillation (AF) refractory to antiarrhythmic drug therapy, catheter or (minimally invasive thoracoscopic) surgical ablation is recommended [1]. Thoracoscopic surgical AF ablation consists of pulmonary vein isolation (PVI) with a minimally invasive epicardial approach with or without additional atrial lesions [2]. Absence of AF after the thoracoscopic approach ranges from 70 to 80% [3, 4]. As thoracoscopic surgical AF ablation generally comprises more extensive lesions than catheter ablation, complications such as sinus node dysfunction (SND) have been described, also in procedures restricted to the left atrium [3, 4]. Similarly, the more invasive surgical Cox Maze III and IV procedures are criticized for high postoperative pacemaker implantation rates. A rate of 8% postoperative pacemaker implantation was previously reported [5, 6].

Irrespective of the invasive treatment employed, SND is associated with AF itself. SND and AF share a mutual pathophysiology, consisting of electrical remodeling and regional fibrosis [7–9]. Regional fibrosis causes conduction slowing and increases repolarization time [10, 11]. SND may also be a pre-existing condition masked by AF episodes, and SND has been described to affect one-fifth of AF patients [11]. Ablation of the left and/or right atrium may further promote or uncover SND. On the other hand, SND is suggested to be a direct consequence of invasive AF ablation, namely, through direct injury or necrosis of the sinus node or the sinus node artery or through atrial edema [12].

Besides PVI and additional left atrial lesions, ganglion plexus (GP) ablation may be a potential additional target to improve AF ablation outcome [13]. The Atrial Fibrillation Ablation and Autonomic Modulation via Thoracoscopic Surgery (AFACT) study included advanced AF patients, undergoing surgical AF ablation, and randomized patients to additional GP ablation or no GP ablation [4]. The AFACT study showed no beneficial effect of additional GP ablation at the cost of a higher procedure-related complication rate. Aside from more major bleedings in the GP group, more pacemaker implantations were found. However, the duration of postoperative SND and actual severity with consequent need for pacing remain unclear, possibly leading to unnecessary pacemaker implantation.

Hence, the objective of the secondary analysis of the AFACT study was to determine the incidence of postoperative SND as well as duration of symptoms in patients who underwent thoracoscopic surgical ablation. We aimed to determine the rate of temporary and permanent pacemaker implantation and the clinical course of severe SND objectified by pacing percentage during follow-up.

## Methods

The AFACT study was a single-center, prospective, randomized trial comparing the efficacy and safety of GP ablation in addition to PVI in AF patients undergoing thoracoscopic surgical AF ablation. The study was registered at clinicaltrials.gov (NCT01091389) and approved by the IRB of the Amsterdam University Medical Centers. All patients with an indication for thoracoscopic surgical ablation were asked for informed consent. In case of consent, a written informed consent was provided. The study enrolled patients between April 2010 and January 2015. The inclusion and exclusion criteria were published before [4]. In brief, the study encompassed patients (60 ± 8 years old) diagnosed with advanced AF, with a long history of AF; with paroxysmal or persistent AF; and with enlarged left atria or previously failed catheter ablation, undergoing thoracoscopic surgical ablation. Main exclusion criteria were prior catheter ablation within the preceding 4 months and NYHA class IV heart failure symptoms. The main trial results have been published before [4].

### Thoracoscopic surgical AF ablation

Preoperative care was standardized in all patients, including left atrial anatomy assessment by non-triggered MRI angiography and transthoracic echocardiography and coronary artery disease assessment by cardiac stress treadmill test (followed by a coronary angiogram when appropriate). All patients were adequately anticoagulated with a vitamin K antagonist or non-vitamin K oral anticoagulant for ≥ 4 weeks prior to surgery.

All patients were subjected to bilateral thoracoscopic PVI on the beating heart with general anesthesia, Supplemental Table 1 [2]. PVI consisted of ≥ 6 radiofrequent (RF) applications to the pulmonary vein (PV) antrum with the Atricure Isolator® Synergy™ bipolar RF ablation clamp. Persistent AF constituted an indication for additional left atrial ablation lines, conforming to the Dallas lesion set, which consists of a superior and trigone line (Atricure Isolator™ Transpolar™ pen or Coolrail® Linear pen) [14, 15]. The left atrial appendage (LAA) was excised using a stapler device. At the time of opening of the pericardium, patients were 1:1 randomized to either additional ablation of the four major GPs and Marshall’s ligament (GP group) or no additional GP ablation (control group) on top of the above-described ablation strategy. Evoked vagal responses by high-frequency stimulation were tested before and after GP ablation in all patients by an electrophysiologist in the operation theater. GPs were localized based on anatomical landmarks as well as based on high-frequency stimulation evoked response [2, 4]. In all patients, entry and exit blocks across PV lines and bidirectional block across left atrial lines were assessed as described previously [16].

### SND diagnosis

In this analysis, SND was defined as symptomatic or asymptomatic junctional escape rhythm with a rate exceeding that of underlying sinus bradycardia or prolonged sinus pauses (≥ 3.0 s) [7]. This is a more loose but also a more comprehensive definition than used in the main publication [17]. Symptomatic SND was defined as a need for admission to the intensive or cardiac care unit, requirement of isoprenaline infusion, or (temporary) pacemaker implantation at the physician’s discretion. All incidences of SND occurring within 30 days postoperatively were included in this analysis. Patient records, procedural logbooks, and all rhythm monitoring (preoperative, perioperative, and postoperative ECG and 24-h Holter) were prospectively obtained. These were assessed by two independent investigators (JN and SO) to determine SND cases for the current analysis.

### Data collection

Baseline patient characteristics, procedural characteristics, and follow-up data were prospectively obtained according to the standardized study protocol [4]. Patients included in the trial were prescribed different pharmacologic treatments prior to the surgery, which failed to relieve AF symptoms. Their medication was continued until the procedure. The day after surgery, patients received their personally prescribed drugs, including antiarrhythmic drugs (AAD). In case of SND, any heart rate-lowering medication, this includes antiarrhythmic drugs, was stopped, but could be restarted once SND was resolved. The latter was discontinued in all patients 3 months postoperatively and could be restarted in cases of recurrent symptomatic AF based on physician’s discretion.

Perioperative ECG was monitored continuously. SND was assessed 1) directly after confirmation of PVI ablation and 2) at the end of surgery. Two times three random complexes were used to measure PR and RR intervals, from which an average interval was calculated (JN). For assessment of consistency, an independent investigator (SO) also assessed 25% of the perioperative ECGs. During admission, continues rhythm monitoring was performed in the recovery room and cardiac care unit. Furthermore, ECGs were performed daily in the ward and before discharge. Patients were followed for rhythm monitoring for which ECGs and 24-h Holters, and these were assessed 3 and 6 months after surgery. Patients were encouraged to obtain additional rhythm recording when symptomatic, and all recorded ECG and Holter data from referral hospitals were collected. This was used to assess recovery to sinus rhythm in patients with SND.

Furthermore, isoprenaline infusion and temporary or permanent pacemaker implantation during or after admission were assessed. The indication for pacemaker implantation was based on treating physician’s discretion. Pacemaker interrogations were performed periodically and entailed analysis of pacing threshold, impedance, and battery endurance. Furthermore, the lower rate and the pacing percentage were collected at the outpatient clinic visit 1 year after permanent pacemaker implantation.

### Fibrosis quantification

The percentage of collagen in the slices of the excised LAA was assessed as a presumed representation of left atrial fibrosis and presumed to be related to fibrosis in SND [18]. Excised LAAs were fixed in 4% formalin and embedded in paraffin. Five-micrometer slices were prepared and stained with Picrosirius red for interstitial collagen quantification. Slices were digitized at × 40 magnification (Philips IntelliSite Ultra Fast Scanner, 0.25 μm/pixel). A random sample of up to 20 non-overlapping fields was selected (4688 × 4521 pixels) from each digitized image. Endocardial, epicardial, and perivascular fibrosis were manually excluded. The ImageJ software color deconvolution was used to automatically determine the area of collagen and the area of cardiomyocytes after exclusion of white background. Collagen area fraction was defined as the area of collagen divided by the combined area of cardiomyocytes and collagen and was averaged over the 20 random fields taken.

### Statistical analysis

Baseline characteristics of patients with and without SND were compared. For the comparison of normally distributed, continuous variables, an unpaired sample *t* test was used; results were expressed as means ± standard deviations (SD). In case of not normally distributed, continuous variables, the Mann-Whitney *U* test was used; results were expressed as median with interquartile range (IQR). Categorical variables were expressed as frequencies with percentages and were compared with the Pearson *χ*^2^ test. Clinical parameters associated with SND incidence were assessed by univariable logistic regression models. The odds ratios (OR) with corresponding 95% confidence intervals (95% CI) were calculated.

The inter-observer variation of perioperative ECG measurements was assessed by calculation of the two-way mixed intraclass correlation coefficient (ICC) and expressed as correlation coefficients.

Data analysis was performed by means of IBM SPSS Version 25 and R version 3.3.2 for Windows (R Foundation for Statistical Computing, Vienna, Austria). A two-sided *p* value of < 0.05 was considered to be significant.

## Results

The AFACT study included 240 patients, of whom, 17 (7.1%) patients developed SND postoperatively, including 15 (6.3%) symptomatic SND cases. Five (29.4%) patients were female, a proportion similar to the 60 (26.9%) patients without SND, *p* = 0.82.

### Demographic characteristics

**Fig.1:**
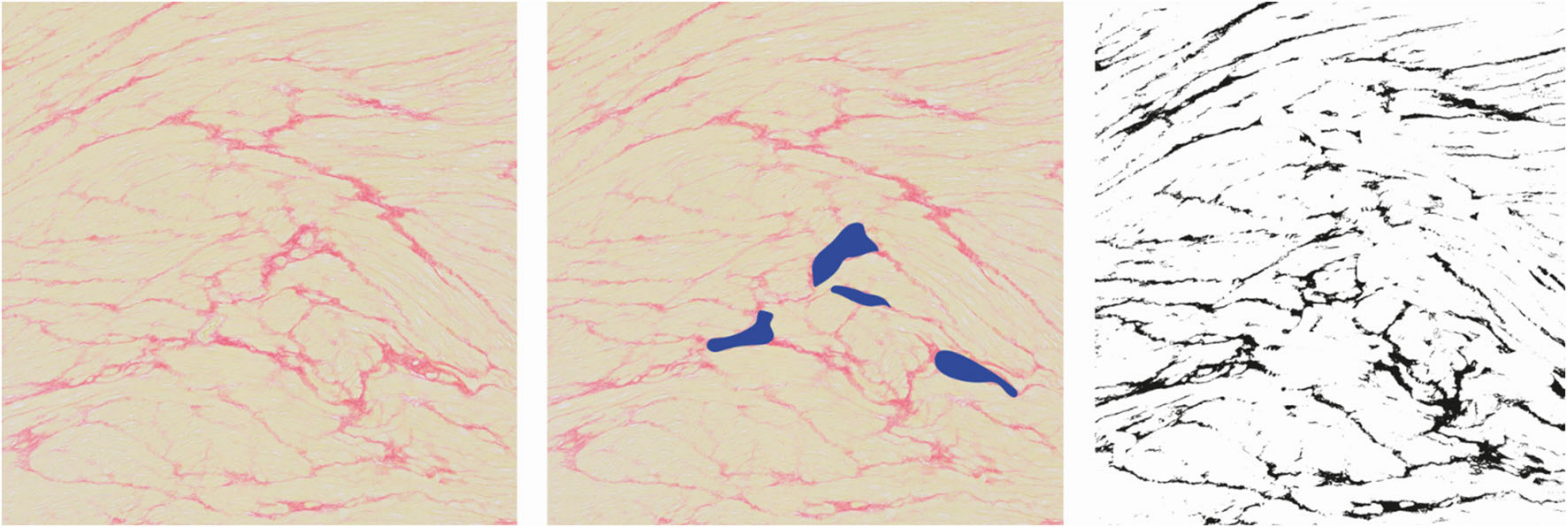
**a** Snapshot of fibrosis in red collagens and in yellow myocardium. Between 15 and 20 snapshots were taken per slice. **b** Manual exclusion of perivascular and endocardial (not seen) fibrosis. **c** Color deconvolution before quantification

Mean age at time of procedure was not significantly different, 61.5 ± 7.8 years in SND patients vs 58.7 ± 8.2 years in patients without SND, *p* = 0.18, Table 1. Fifteen (88.2%) patients with SND were diagnosed with persistent AF compared to 128 (57.4%) patients without SND, *p* = 0.01. The rate of AAD prescription before the surgery was similar in both groups. This also held true for AV nodal blocking drugs, such as beta-blockers and calcium antagonists; univariable analysis showed an OR 0.64 (95% CI 0.03–3.49, *p* = 0.68). Fibrosis in the left atrial appendage was quantified in seven (41.2%) patients with SND and 102 (45.7%) patients without SND, *p =* 0.72. The percentage of fibrosis was similar between both groups, 14.5% in patients with SND and 15.4% in patients without SND, *p =* 0.82, Fig. 1.

**Table 1:** Baseline characteristics of patients with and without sinus node dysfunction

	SND (*n* = 17)	No SND (*n* = 223)	*p* value
Female, *n* (%)	5 (29.4)	60 (26.9)	0.82
Age, years mean ± SD	61.5 ± 7.8	58.7 ± 8.2	0.18
BMI, kg/m^2^ mean ± SD	27.1 ± 4.1	27.3 ± 3.9	0.77
AF type			0.01
Paroxysmal, *n* (%)	2 (11.8)	95 (42.6)	
Persistent, *n* (%)	15 (88.2)	128 (57.4)	
AF duration, years median (IQR)	3.0 [1.5–5.0]	4.0 [2.0–8.0]	0.18
LA volume index, ml/m^2^ mean ± SD	39.3 ± 13.0	39.4 ± 11.8	0.97
LV ejection fraction, % mean ± SD	49.5 ± 9.0	50.4 ± 9.7	0.73
Previous catheter PVI, *n* (%)	2 (11.8)	54 (24.2)	0.24
Previous MI, *n* (%)	1 (5.9)	10 (4.5)	0.79
Previous PCI, *n* (%)	2 (11.8)	16 (7.2)	0.49
CHA_2_DS_2_-VASc score			0.62
0, *n* (%)	3 (17.6)	64 (28.7)	
1, *n* (%)	6 (35.3)	69 (30.9)	
> 2, *n* (%)	8 (47.1)	90 (40.4)	
Congestive heart failure, *n* (%)	2 (11.8)	10 (4.5)	0.18
Hypertension, *n* (%)	10 (58.8)	94 (42.2)	0.18
Previous CVA, *n* (%)	3 (17.6)	17 (7.6)	0.15
Diabetes mellitus, *n* (%)	1 (5.9)	15 (6.7)	0.89
Vascular disease, *n* (%)	2 (11.8)	23 (10.3)	0.85
Age 65–74 years, *n* (%)	6 (35.3)	62 (27.8)	0.51
Age > 75 years, *n* (%)	1 (5.9)	1 (0.4)	0.02
Antiarrhythmic drugs			
Class IA, *n* (%)	0 (0.0)	6 (2.7)	0.49
Class 1C, *n* (%)	4 (23.5)	75 (33.6)	0.39
Class II, *n* (%)	7 (41.2)	116 (52.0)	0.39
Class III, *n* (%)	9 (52.9)	87 (39.0)	0.26
Class IV, *n* (%)	1 (5.9)	31 (13.9)	0.35
Other, *n* (%)	3 (17.6)	25 (11.2)	0.43
Digoxine, *n* (%)	0 (0.0)	11 (5.5)	0.35
LAA fibrosis (%)	14.5	15.4	0.82

*CVA*, cerebrovascular event; *IQR*, interquartile range; *MI*, myocardial infarction; *PCI*, percutaneous catheter intervention; *PVI*, pulmonary vein isolation; *SD*, standard deviation

*CCU*, cardiac care unit; *ICU*, intensive care unit

### Preoperative rhythm monitoring

No difference in heart rhythm, PR interval, and preoperative heart rates was found on ECG or 24-h Holter monitoring between both groups. Specifically, no difference in minimal heart rate on Holter monitoring was found, *p* = 0.92, Supplemental Table 2. Of note, preoperatively, 13 (76.5%) patients with SND postoperatively were in sinus rhythm and had normal conduction parameters on the ECG.

### Surgery details

All patients underwent thoracoscopic PVI; however, due to periprocedural bleeding, the procedure was terminated early in two patients. In five (5.2%) patients with paroxysmal AF, additional left atrial lesions were constructed, due to high burden of (symptomatic) paroxysmal AF preoperatively. However, in four (2.8%) persistent AF patients, no additional lesions were constructed, which was a violation of protocol. GPs were ablated in 11 (64.7%) SND patients not significantly different to 106 (47.5%) patients without SND, *p* = 0.17, Table 2.

**Table 2:** Ablation and treatment characteristics in patients with and without sinus node dysfunction

	SND (*n* = 17)	No SND (*n* = 223)	*p* value
Additional left atrial lesion set, *n* (%)	16 (94.1)	127 (56.9)	0.003
Ganglion plexus ablation, *n* (%)	11 (64.7)	106 (47.5)	0.17
Admission duration, days mean ±SD	7.5 ± 3.0	5.0 ± 1.8	0.003
Admission ICU or CCU, *n* (%)	13 (76.5)	43 (19.3)	< 0.001
Rhythm during admission, *n* (%)			
Atrial flutter, *n* (%)	4 (23.5)	23 (10.3)	0.10
Atrial fibrillation, *n* (%)	1 (5.9)	0 (0.0)	< 0.001
Isoprenaline administration, *n* (%)	4 (23.5)	n/a	
Temporary pacing, *n* (%)	6 (35.3)	n/a	
Permanent pacing, *n* (%)	6 (35.3)	1 (0.4)	< 0.001

### Perioperative ECG

The inter-observer validity for measuring the PQ interval directly after ablation and at the end of surgery was good (ICC = 0.76, *p* < 0.001 and ICC = 0.61, *p* < 0.001, respectively). The inter-observer validity was very high for measuring the HR directly after ablation and at the end of surgery (ICC = 0.99 *p* < 0.001 and ICC = 0.92, *p* < 0.001, respectively).

Perioperative ECG recordings showed no difference in the PQ interval directly after ablation (186 ± 51.7 vs 189 ± 31.3 ms, *p* = 0.87) or at the end of surgery (195 ± 42.5 vs 190 ± 33.1 ms, *p* = 0.73), Supplemental Table 2. Next, significantly lower heart rates directly after ablation were observed in patients with a diagnosis of SND compared with those without SND (61 ± 11.1 vs 71 ± 13.0 bpm, *p* = 0.01). At the end of surgery, heart rate was lower in patients with a diagnosis of SND (59 ± 12.9 bpm) compared with those without SND (71 ± 15.1 bpm), *p* = 0.003.

### Postoperative admission

**Fig. 2:**
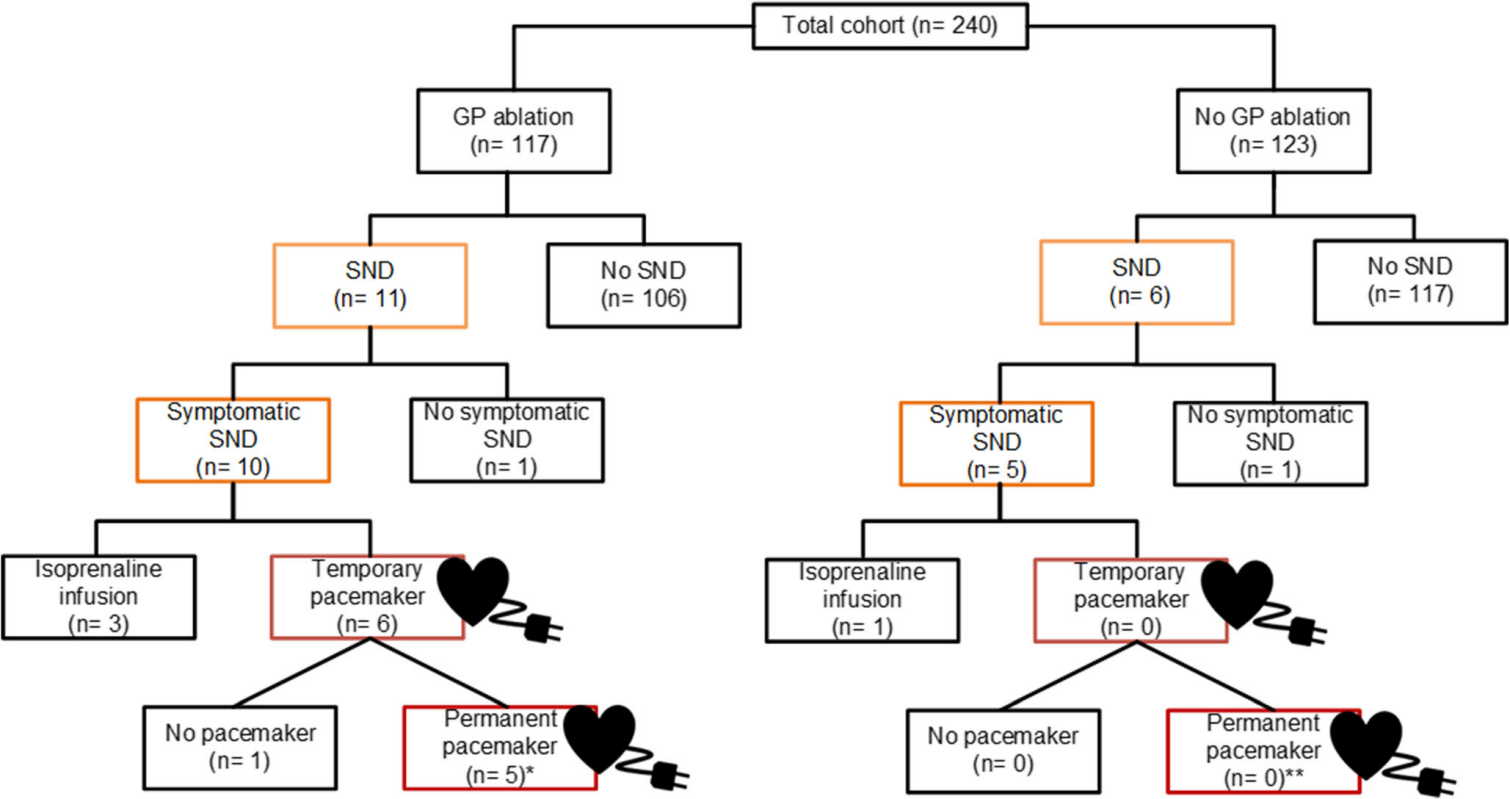
Flowchart of sinus node dysfunction (SND) cases and their treatment after thoracoscopic surgical atrial fibrillation ablation per randomization arm. Additional ganglion plexus (GP) ablation or no additional ablation. *The AFACT study reports six patients; however, one patient was implanted with a pacemaker 1 year after surgery and therefore not considered as due to postoperative SND [4]. **One patients was implanted with a pacemaker few years after surgery and therefore not considered as due to postoperative SND

Patients with SND were admitted significantly longer, mean 7.5 ± 3.0 days, than patients without a SND diagnosis, 5.0 ± 1.8 days, *p* = 0.003, Table 2. More patients with SND (*n* = 13, 76.5%) were admitted to the intensive or cardiac care unit than patients without SND (*n* = 43, 19.3%), *p* < 0.001. Most frequently, patients were admitted for standard hemodynamic observations during the first 24 h postoperative. Four (23.5%) patients with symptomatic SND required isoprenaline infusion, Fig. 2.

### Temporary and permanent pacemakers

**Fig. 3:**
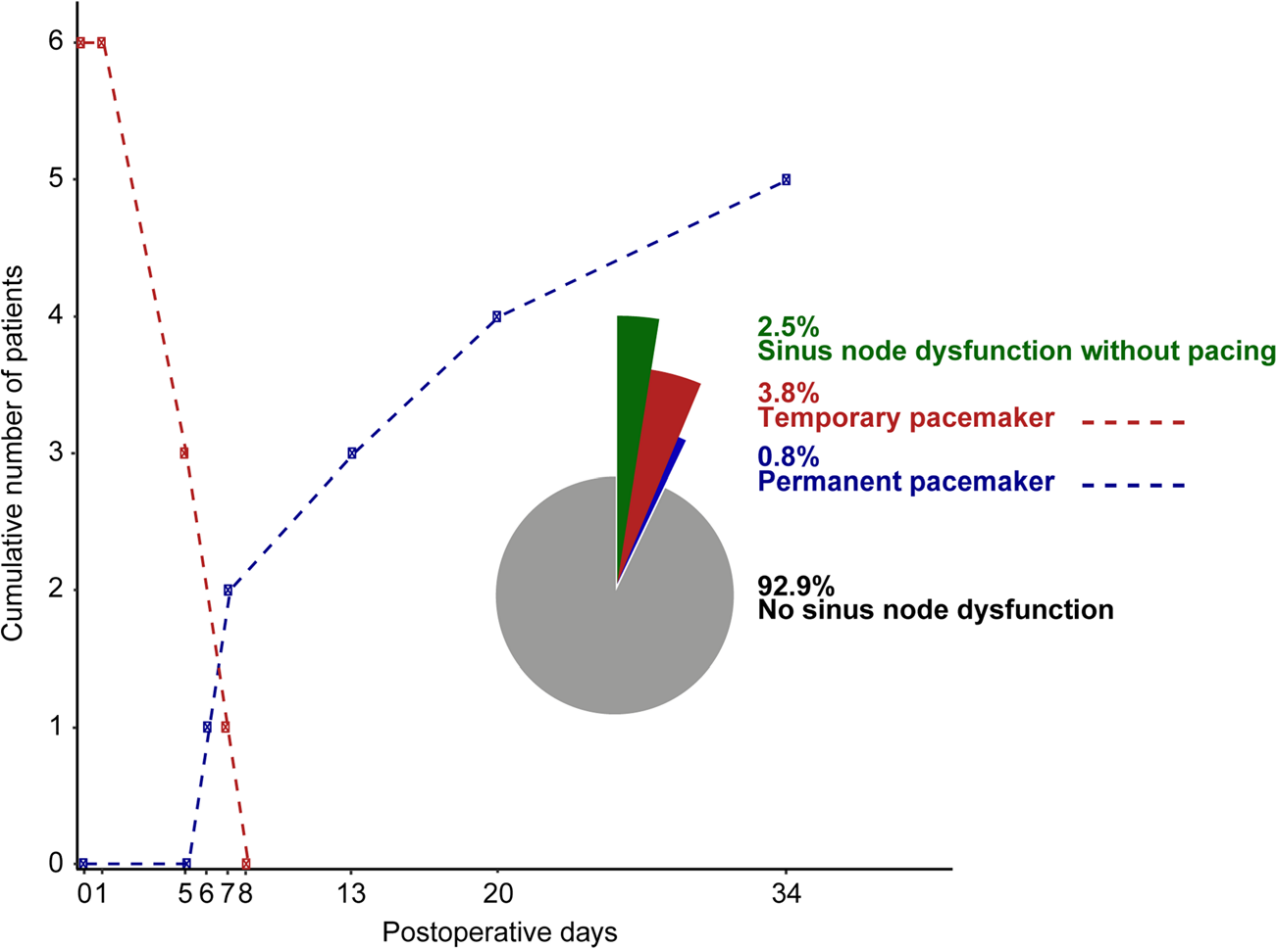
A Percentage of patients with sinus node dysfunction stratified for pacing necessity. Cumulative number of patients with a temporary pacemaker (red) or a permanent pacemaker (blue) implanted for the days postoperative. Pie chart shows the percentage of patients with sinus node dysfunction stratified for pacing necessity

During admission, six (40.0%) of the symptomatic SND cases were paced temporarily due to symptomatic bradycardia, of whom, one patient was also administrated isoprenaline after 1 day due to malpacing of the temporary lead, Fig. 3. Patients were paced for 1 to 7 days.

At 3 months, 93.8% of the patients with postoperative SND were in sinus rhythm, with a mean heart rate of 79.9 ± 10.2 bpm. At 6 months, sinus rhythm was found in 92.9% of the patients with postoperative SND, with a mean heart rate of 75.9 ± 10.9 bpm, respectively. This was similar to the heart rhythm and rate in patients without SND (data not shown). The remaining patients in both groups had an ECG registration of an atrial arrhythmia.

Permanent pacemakers, in DDD mode, were implanted 6 to 34 days postoperatively in five (2.1%) patients, of whom, two (0.8%) patients were not paced during admission, Table 3. These patients remained symptomatic and therefore required a permanent pacemaker. One year after pacemaker implantation, one patient was not paced at all, three (1.3%) patients were (atrially or ventricularly) paced 1–2.5% of the time, and one patient was paced atrially 64% of the time and ventricularly 47% of the time. The lower rate of the pacemakers was set at 50 or 60 beats per minute.

**Table 3:** Case description of patients who received a temporary or permanent pacemaker

	Sex	Age	AF type	Duration temporary pacing (days)	Permanent pacemaker (implanted postoperative day)	Lower rate (bpm)	Pacing at 1 year follow-up (atrial (A) - ventricle (V))
1	Male	59	Persistent	1	n/a	n/a	n/a
2	Male	65	Persistent	7	6	60	A 64% - V 47%
3	Male	56	Persistent	5	n/a	n/a	n/a
4	Male	51	Persistent	1	13	60	A 0% - V 0%
5	Male	68	Persistent	5*	n/a	n/a	n/a
6	Male	59	Paroxysmal	1	7	50	A 2.4% - V 2%
7	Female	63	Persistent	n/a	34	50	A 1% - V 1%
8	Male	67	Persistent	n/a	20	50	A 15% - V 12%

*Patient suffered from bradycardia directly postoperatively; however, there was malpacing of temporary pacemaker; therefore, it was treated with isoprenaline infusion

### Risk factors of sinus node dysfunction

**Fig. 4:**
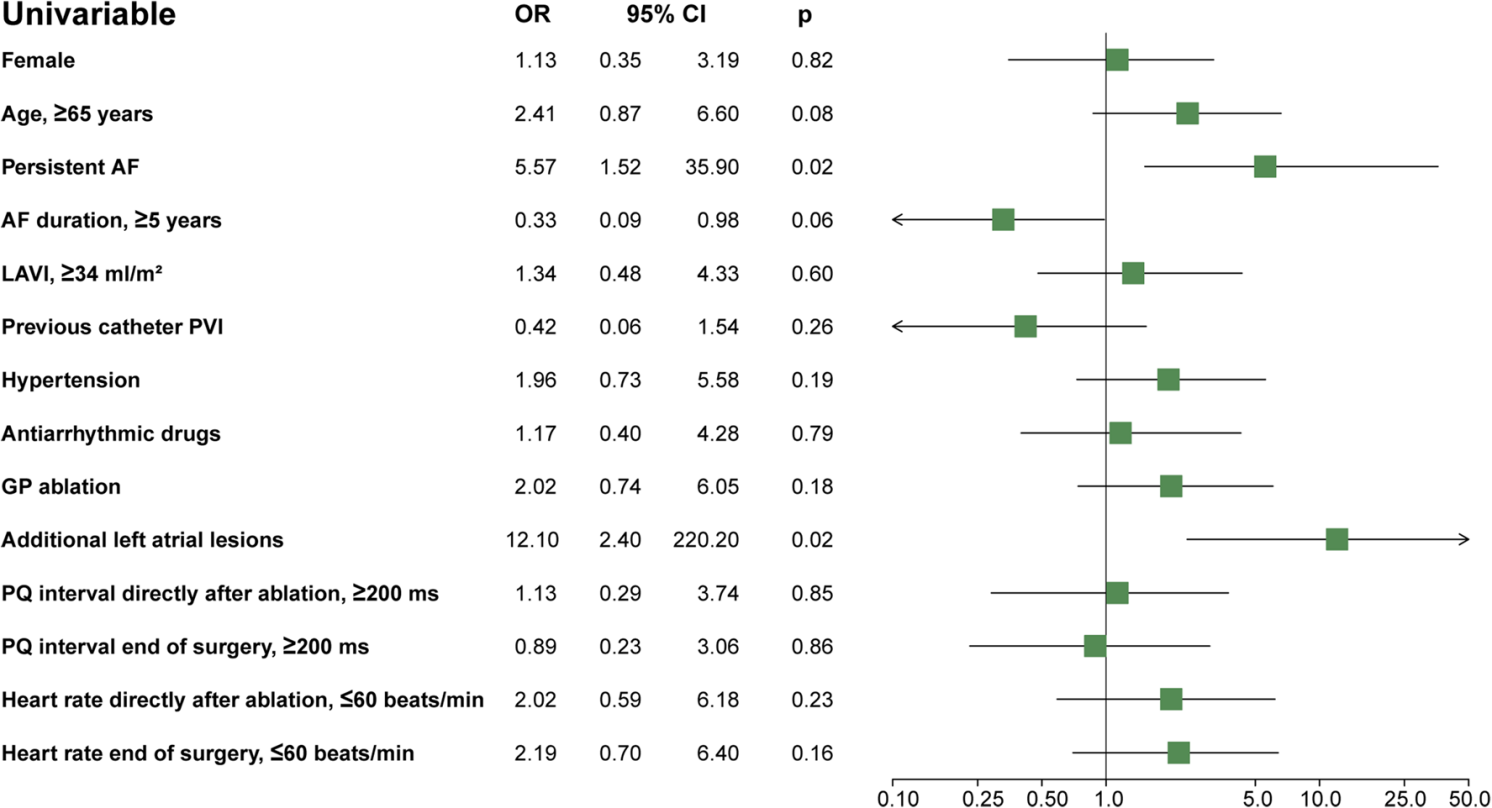
Forest plot of the univariable odds ratios of risk factors of sinus node dysfunction after thoracoscopic surgical AF ablation. Patients were randomized to additional ganglion plexus (GP) ablation or no additional GP ablation. AF, atrial fibrillation; LAVI, left atrial volume index; OR: odds ratio; p, *p* value; PVI, pulmonary vein isolation; 95% CI, 95% confidence interval

Univariable analyses showed that persistent AF (OR 5.57 CI 1.52–35.90, *p* = 0.02) and additional left atrial ablations (OR 12.10 CI 2.40–220.20, *p* = 0.02) were associated with postoperative SND, Fig. 4. Importantly, additional left atrial lesions were indicated in patients with persistent AF, however were not performed in all patients with an indication (reasons listed above). The number of SND patients was too low for an extensive multivariable analysis.

## Discussion

We found that SND, defined as a symptomatic or asymptomatic junctional rhythm with a frequency exceeding sinus bradycardia or prolonged sinus pauses (≥ 3.0 s), occurred in 7.1% of patients after thoracoscopic ablation of AF in patients randomized in the AFACT trial. Importantly, the majority of SND was of temporary nature and resolved to a normally conducted sinus rhythm within days postoperatively. The occurrence of SND was not significantly different in patients randomized to GP ablation or control. The ablation of additional left atrial lesions was a very strong risk factor to develop SND postoperatively. A large proportion of SND cases was symptomatic and frequently required isoprenaline infusion or temporary pacing. At 1 year, we found a very low pacing percentage.

### Surgical ablation may cause SND

Additional atrial ablations were found to increase the risk of SND strongly. This suggests that the surgery itself is directly instrumental in the development of SND. Indeed, thoracoscopic surgical AF ablation has been found to directly cause SND postoperatively due to the scaring of the atrium [12]. The ablation gives rise to local inflammation and edema and may also injure the sinus node or the sinus nodal artery [12]. The sinus node artery mostly originates from the right coronary artery, but has been described to originate from the circumflex artery in 40% of the patients [19]. In that case, the artery courses through the field that is ablated, as it winds around the PVs. Consequently, more extensive atrial ablations may increase the risk of sinus node via direct or indirect injury. Since preoperative coronary angiography was not standard of care, we were able to assess the origin of the circumflex artery by coronary angiographies retrospectively in 29.4% of the patients with and 14.8% of the patients without SND. In this small subgroup analysis, the origin was not significantly different. Due to the sample size, it should be valued as hypothesis generating and demands prospective assessment in a larger cohort. The fact that SND resolved spontaneously a few days postoperatively in the majority of the patients suggests a direct role of edema or injury to the sinus node artery or other structures, rather than permanent damage.

The original cut-and-sew Cox Maze surgery, consisting of biatrial lesions, is more invasive than the thoracoscopic surgical approach. Kolikov et al. showed that prior to the Cox Maze III surgery, 13% of AF patients suffered from SND during electrophysiology studies. These patients received a pacemaker postoperatively. Furthermore, 9% received a pacemaker for a nodal rhythm with a low heart rate and pauses lasting more than 3 s [12]. These patients corresponded to our definition of SND, but these numbers may be biased by 1) the unknown number of patients with postoperative SND without a pacemaker or 2) a low threshold for implantation potentially due to under appreciation of the temporary nature of SND in this setting. In comparison, we report that 2.1% of the patients received a permanent pacemaker.

### Atrial myopathy

Aside from direct, procedure-related damage, SND and AF may interact with each other and probably share a mutual atrial myopathy [7–9]. Sanders et al. found diffuse atrial anatomical and structural abnormalities leading to conduction slowing and delay in SND patients [7, 20]. Furthermore, it was found that calcium handling is altered in SND patients, which is also found in AF patients [7, 21]. These results argue that SND and AF share a similar atrial myopathy, which may lead to an increased susceptibility for the incidence of both.

Next, increased (regional) fibrosis has been described in SND patients, causing conduction slowing and increasing repolarization time [10, 11]. Previous studies have shown that fibrosis is a progressive process and may be associated with the progression of AF [22]. Extensive fibrosis of both right and left atria on late enhancement MRI is associated with pacemaker necessity [9]. It is likely that fibrosis is more outspoken in patients with SND, since they more frequently suffer from persistent AF. However, in a subgroup analysis of fibrosis quantification in the LAA, which was used as for a proxy of left atrial fibrosis, we found a similar percentage of interstitial collagen in patients with and without SND. Notably, patients undergoing surgical ablation are symptomatic patients with AF refractory to antiarrhythmic drugs. The clinical distinction between paroxysmal and persistent AF may not fully comprehend the distinction on tissue level, especially in patients with these advanced forms of AF. These findings need to be confirmed in a larger cohort.

### Indication for pacemaker treatment

We merely found five (29.4%) patients with SND being acutely implanted with a permanent pacemaker. Importantly, at 1 year, we found low pacing percentages. Moreover, 90% of patients diagnosed with SND were in sinus rhythm with normal sinus frequencies on Holter during follow-up. This implies that SND is indeed temporary in most cases, as it frequently resolves within days. A wait-and-see approach of 1–2 weeks may therefore be the most convenient. During this wait-and-see period, a symptom-driven, stepwise medical approach should be installed, including daily rate and rhythm controls. Any heart rate-lowering medication should (temporarily) be discontinued, and isoprenaline infusion may be administered to patients who remain symptomatic. In case of refractory symptoms, a temporary pacemaker may be implanted. Of note, this stepwise approach should be evaluated for each individual case. Taken together, this would imply prolonging the clinical admission until the heart rate has resumed sufficiently for discharge. Future studies may assess feasibility of a semi-permanent pacemaker that can easily be extracted after a few weeks or months. This may assist patients who remain symptomatic after 1–2 weeks, but are likely not to need pacing after a few months.

### Limitations

Preoperative SND was assessed by Holter monitoring and ECG, instead of continues rhythm monitoring, which may have underestimated the number of patients with SND. Of note, a considerable proportion of the patients were in AF, and pre-existing SND may have been masked. However, at baseline, more than 75% of the patients were not in AF, thus potentially, pre-existing SND could be diagnosed.

We could not determine the amount of fibrosis in the LAA in the total cohort due to sampling errors. However, the analyzed sample was a random sample and therefore is not expected to have influenced the results.

## Conclusion

These findings suggest that SND following thoracoscopic ablation for AF is related to a surgical complication, rather than to unmasking of pre-existing SND after elimination of AF. Importantly, the majority of postoperative SND was transient and resolved to a normally conducted sinus rhythm within days postoperatively. This indicates that a conservative approach with regard to pacemaker implantation in patients with SND after thoracoscopic surgical ablation for advanced AF should be recommended.

## Electronic supplementary material

ESM 1 (DOCX 18 kb)
