# Supplementary material for: Clinical course of sinus node dysfunction after thoracoscopic surgery for atrial fibrillation—analysis of the Atrial Fibrillation Ablation and Autonomic Modulation via Thoracoscopic Surgery (AFACT) study
Source: J Interv Card Electrophysiol. 2020 Mar 14;60(2):185–93. doi: 10.1007/s10840-020-00722-0 (PMC7925456; doi:10.1007/s10840-020-00722-0)
Supplement: Supplementary file 1 — (DOCX 18 kb) [file 10840_2020_722_MOESM1_ESM.docx]

| **Start of surgery** | Thoracoscopic surgical atrial fibrillation ablation was performed on the beating heart under general anesthesia. |
| --- | --- |
|  | A double-lumen endotracheal tube for selective lung ventilation was placed. |
|  | On the patient's right side two 10-mm ports in the fourth and sixth intercostal space midaxillary line, and one port in the third or fourth intercostal space anterior axillary line were placed. |
|  | Epicardium was opened and cardiac anatomy revealed. |
| **Ganglion plexus** | Ganglion plexi (GP) were localized with high-frequency stimulation (18 V, 1-ms pulse width, 1000 Hz) on the anterior right GP and inferior right GP by an Atricure Cooltip (Atricure Inc, Cincinnati, OH) ablation pen positioned on the fat pad containing the GP and connected to an external pacemaker device, Oscor Pace 203H DDD External Dual-Chamber Pacemaker (Oscor Inc, Palm Harbor, FL). |
|  | Positive response was confirmed when high-frequency pacing induced AV block or increased the R-R interval >50%. |
| **Ablation** | GPs were ablated with bipolar radiofrequent energy through the AtriCure Cooltip pen, when randomized to the GP ablation arm. |
|  | A rubber band was placed was under the pulmonary venous (PV) antrum using a lighted dissector (AtriCure Lumitip Dissector) after blunt dissection of the Waterstone groove. |
|  | An AtriCure Isolator Transpolar Clamp was then connected to the rubber banding and positioned gently around the PV antrum. |
|  | Pulmonary vein isolation (PVI) was constructed by application of bipolar radiofrequency energy to the clamps around the PV antrum. |
|  | PVI was confirmed for entry and exit block with local electrograms around the ablation scar. Electrograms were interpreted using a mobile electrophysiological work station (Bard Labsystem PRO 2.4A, C. R. Bard Inc). |
|  | The procedure was repeated on the left side of the thorax. |
|  | The ligament of Marshall was dissected and ablated in all patients. |
|  | Additional left atrial lines were constructed in patients with persistent AF and consisted of a superior line (SL) and trigone line (TL)*.* |
|  | SL connects the ablation lines around the left and right pulmonary veins. |
|  | TL connects the SL and the left fibrous trigone. |
| **End of surgery** | After the ablation procedure and confirmation of conduction block, the LAA was removed with an endoscopic stapling device (Endo Gia stapler, Tyco Healthcare Group, North Haven, CT). |
|  | On completion of the procedure, single chest drains were inserted; one in each side of the chest. |

Supplemental table 1. Procedural step of thoracoscopic surgical atrial fibrillation ablation. Krul et al. (Circ AE 2011) further described the surgery in detail.

|  | SND  (n= 17) | No SND  (n= 223) | P-value |
| --- | --- | --- | --- |
| Preoperative ECG: |  |  |  |
| Heart rhythm: |  |  | 0.88 |
| Sinus rhythm, n (%) | 13 (76.5) | 191 (85.7) |  |
| Atrial flutter, n (%) | 0 (0.0) | 5 (2.2) |  |
| Atrial fibrillation, n (%) | 0 (0.0) | 16 (7.2) |  |
| Heart rate, bpm mean±SD | 62.8±17.4 | 63.3±15.8 | 0.91 |
| PR interval, ms (SD) | 184±23 | 175±32 | 0.28 |
| Preoperative 24h Holter: |  |  |  |
| Heart rhythm: |  |  |  |
| Atrial tachycardia, n (%) | 2 (15.4) | 23 (11.2) | 0.85 |
| Atrial flutter, n (%) | 3 (23.1) | 37 (18.0) | 0.65 |
| Atrial fibrillation, n (%) | 8 (61.5) | 100 (48.8) | 0.37 |
| Heart rate min, bpm mean±SD | 46.3±10.6 | 46.6±9.9 | 0.92 |
| Heart rate average, bpm mean±SD | 73.4±20.7 | 74.5±17.3 | 0.83 |
| Heart rate max, bpm mean±SD | 140.5±43.7 | 135.6±37.5 | 0.65 |
| Perioperative ECG directly after ablation: |  |  |  |
| PQ interval, ms±SD | 186±51.7 | 189±31.3 | 0.87 |
| Heart rate, bpm mean±SD | 61±11.1 | 71±13.0 | 0.01 |
| Perioperative ECG end of surgery: |  |  |  |
| PQ interval, ms±SD | 195±42.5 | 191±33.1 | 0.66 |
| Heart rate, bpm mean±SD | 59±12.9 | 71±15.1 | 0.003 |

Supplemental table 2. Preoperative and perioperative ECG and Holter characteristics. bpm: beats per minute, ms: milliseconds.
